# 4237 TL1 Team Approach to Peripartum Obsessive-Compulsive Disorder: a meta-analysis of the perceived impact of gestation and delivery on symptomology

**DOI:** 10.1017/cts.2020.154

**Published:** 2020-07-29

**Authors:** Danielle Laine Cooke, Rebecca Henderson, Joseph McNamara, Carol Mathews

**Affiliations:** 1 University Of Florida Clinical and Translational Science Institute; 2 University of Florida, College of Medicine

## Abstract

OBJECTIVES/GOALS: Obsessive compulsive disorder (OCD) is a serious and impairing disorder. The peripartum is associated with changes in pre-existing OCD, including exacerbation and improvement of the disorder. This meta-analysis seeks to understand the proportion of women reporting a change in OCD during this time. METHODS/STUDY POPULATION: Nine studies with independent samples examining change in obsessive-compulsive symptomology (OCS) in the peripartum were included in the meta-analysis. Studies were included if the sample examined with women with a clinical diagnosis of OCD that pre-existed pregnancy onset. The meta-analysis was conducted using R Studio with Meta, Metafor and Weightr packages. A moderation analysis was conducted to examine the impact of gestational period on OCD symptoms. Gestational periods were defined as pregnancy, postpartum, or the peripartum. Peripartum refers to a collapsed postpartum/pregnant period such that the period was not identified or specified during data collection. RESULTS/ANTICIPATED RESULTS: The summary proportion of women who experienced no change in symptoms was 46.7% (CI: 42.0-51.4%). No change by period was: pregnancy 49.6% (CI: 36.3-62.9%); postpartum 45.6% (CI: 41.4-49.9%); peripartum 52.4% (CI: 42.4-50.3%). The summary proportion of women who experienced exacerbation was 39.2% (CI: 33.5-45.5%). Exacerbation by period: pregnancy 35.5% (CI: 24.8-47.9%); postpartum 42.9% (CI: 34.8-51.4%); peripartum 34.6% (CI: 23.7-47.4%). The summary proportion of women who experienced improvement was 11.5% (CI: 9.3-14.4%). Improvement by period: pregnancy 42.9% (CI: 14.7-77.0%); postpartum 7.8% (CI: 5.7-10.4%); peripartum 19.6% (CI: 13.7-27.3%). Gestational period had a moderating effect. DISCUSSION/SIGNIFICANCE OF IMPACT: During the peripartum 46% report no change, 40% a worsening and 12% an improvement. Improvement typically occurs during pregnancy and may be followed by a postpartum worsening. This may reflect a hormonally-sensitive subsection of women impacted by the acute changes that occur during this time.

